# Supplementary material for: Clinical and pathological features of thrombotic microangiopathy influencing long-term kidney transplant outcomes
Source: PLoS One. 2020 Jan 10;15(1):e0227445. doi: 10.1371/journal.pone.0227445 (PMC6953866; doi:10.1371/journal.pone.0227445)
Supplement: Attachment — Submitted filename: RESPONSE_TO_REVIEWERS.docx [file pone.0227445.s001.docx]

**Dr. Cínthia Montenegro Teixeira**

Nephrology Division

Hospital do Rim – Fundação Osvaldo Ramos - Universidade Federal de São Paulo, São Paulo, Brazil

Email address: [cinthiamt@gmail.com](mailto:laila_viana@hotmail.com)

November 1^st^, 2019

**Subject:** Response to reviewers and Editor

Giuseppe Remuzzi, Academic Editor
PLOS ONE

Dear Academic Editor and reviewers,

Please find enclosed the corrected version of the manuscript entitled “Clinical and pathological features of thrombotic microangiopathy influencing long-term kidney transplant outcomes”. The answers to the reviewers are disposed bellow.

1. **need to expand more the conclusions**

It is important to recognize that the lack of a tailored therapeutic strategy, as the complement blockade, partially accounts for the bad outcomes of our findings. Further prospective studies carefully looking at the impact of this treatment are required to test this hypothesis.

This statement was added on “Discussion” section.

2. **need to discuss about the high rate of allograft loss and potential causes of this**

The high rate of renal allograft loss in our cohort is probably related to the unspecific therapeutic approach performed, explained by the unavailability of complement blockers and diagnostic tools for differentiation of the etiologies and triggers of post-transplant TMA.

This statement was added on “Discussion” section.

3. **need to clarify if dialysis was temporary**

At the time of TMA diagnosis, 36% of the patients (n=32) were on dialysis. There was allograft loss within the first 3 months post transplant in 13 patients.

This information was written in “Results” section.

4. **concern about the statement that their cohort suggest risk factors for development of *de novo* TMA**

We caution that, due the high proportion of patients with primary kidney disease of unknown etiology in our data, we cannot fully ascertain whether some cases of recurrent aHUS were misdiagnosed as *de novo* TMA.

We added this caveat on “Discussion” section and suppressed the expression “*de novo*” of the sentence on line 457.

5. **need to implement the discussion about eculizumab therapy and its use in Brazil, if any.**

Although eculizumab was not a therapeutic option in our cohort, since, in Brazil's publicly funded health care system, it is only available for treatment of patients with paroxysmal nocturnal hemoglobinuria (PNH). The high cost of medication and need for prolonged treatment preclude the financing of this therapy by the local transplant centers or the patient itself.

We addressed this issue on “Discussion” section.

6. **Review Line 71 and Line 510: I would change the verb "prove" to "suggest" in the conclusion.**

Thank you. We changed the verb.

7. **In the methods section, they should include how the cases were reviewed for the diagnosis of TMA prior to the confirmation by reviewing kidney biopsies, and include the methods of their search such as "CPT codes" used or explained how they found/ collected 119 cases with diagnosis of TMA (how was this original diagnosis made).
   And this should be included as a limitation in the discussion part.**

In Brazil or Hospital do Rim, the search for CPT codes is not available. In this retrospective cohort study, we initially retrieved all consecutive unselected reports of renal transplant biopsies from Hospital do Rim database between January 2011 and December 2015. These biopsies were performed for graft dysfunction, new onset proteinuria or delayed graft function from kidney and kidney-pancreas transplanted patients. Of a total of 6886, we selected 119 biopsies whose reports described features of TMA. Final diagnosis was confirmed by one of the pathologist authors (LARM).

We added this information on “Methodology” section. In addition, we wrote a statement on “Discussion” section addressing the possible selection criteria bias as a limitation of our study, since TMA diagnosis was based only on histological criteria.

8. **Please include a copy of Table 5 which you refer to in your text on page 18**

On page 18 line 367, there was a typing error: there is no Table 5. The results are related to Table 4, instead. We are sorry about it.

9. **Additional requirements**

We amended current and reviewed statements about ethics and competing interests in the manuscript and in the online submission form.

I hope this new version will fit the requirements for publication in PLOS ONE.

I am looking forward to hearing from you soon.

Sincerely,

Dr. Cínthia Montenegro Teixeira, the corresponding author.
